# The relationship between photo retouching addiction and social appearance anxiety for social internet users

**DOI:** 10.3389/fpubh.2025.1594396

**Published:** 2025-06-11

**Authors:** Desheng Yan, Guangming Li

**Affiliations:** ^1^Inner Mongolia Minzu Preschool Education College, Ordos, China; ^2^Key Laboratory of Brain, Cognition and Education Sciences (South China Normal University), Ministry of Education, Guangzhou, China; ^3^School of Psychology, Center for Studies of Psychological Application, and Guangdong Key Laboratory of Mental Health and Cognitive Science, South China Normal University, Guangzhou, China

**Keywords:** photo retouching addiction scale (PRAS), social appearance anxiety, selfie addiction, social internet users, photo retouching addiction

## Abstract

There is a large population of social internet users who habitually retouch photos. These behaviors may be harmful, as some studies have shown that photo retouching behaviors may result in the potential risk of dependency and some addiction-like symptoms. Specifically, chronic photo retouching behaviors of social internet users can lead to negative emotions, low satisfaction with appearance, and non-essential cosmetic surgery. Severe cases may suffer from “Snapchat dysmorphia” which could become a form of body dysmorphic disorder. In this paper, the photo retouching addiction scale (PRAS) was developed as a psychometric instrument to measure the degree of photo retouching addiction for social internet users. Through a cross-sectional study, the social appearance anxiety scale (SAAS) was combined with the PRAS to investigate whether the relationship exists between photo retouching addiction and social appearance anxiety. Results show that: (1) The developed PRAS includes five dimensions and has good reliability and validity (Cronbach’s Alpha = 0.862; χ^2^/*df* = 1.511, CFI = 0.975, TLI = 0.968, RMSEA = 0.041, SRMR = 0.031); (2) The photo retouching addiction is significantly positively correlated with social appearance anxiety; (3) Sex, age, and marital status of social internet users have a significant impact between photo retouching addiction and social appearance anxiety.

## 1 Introduction

As an increasing number of social internet users have begun to post retouched photos on social network sites (SNS), the potential risks of these behaviors are being discussed more (1). Young people manage their body image by relying on appearance-ideals conveyed by social media and such use of social media maintains and/or reinforces existing body image issues (2). According to the Meitu Image Life Report (3), there are more than 240 million active social internet users in China per month, with more than seven billion retouched pictures and video production per month. From report of FaceTune (4), a photo retouching software had a 20% increase in usage and exported 1–1.5 million retouched photos per day.

The negative body image may mediate this association, highlighting a potential pathway through which social withdrawal influences social media use patterns (5). The photo retouching behaviors can be addictive and harmful as follows: Firstly, the photo retouching behaviors may lead to a decrease in personal appearance satisfaction for social internet users, which is associated with increased negative emotions. The higher the level of photo retouching is, the higher the level of self-objectification compared with appearance is and the lower the appearance satisfaction is (6, 7). Secondly, the photo retouching behaviors may occur in conjunction with eating disorders for social internet users. The social media has been implicated as a correlate or cause of increased disordered eating in men and women (8). Thirdly, the photo retouching behaviors may lead to the risk. Some social internet users become dependent on these photo retouching behaviors. Excessive photo retouching behaviors can be associated with several addiction-like symptoms such as dependence and difficulty in parting with photo retouching software (9). Last but not least, the use of photo retouching software increases the acceptance of plastic surgery (10). Due to too many photo retouching behaviors displayed on social media, some photo retouching addicts undergo non-essential cosmetic surgery (11).

In 2019, 72% of members of the American academy of facial plastic and reconstructive surgery reported some patients of social internet users who sought cosmetic surgery to improve their selfies (11). Severe cases may suffer from the “Snapchat dysmorphia” which has sparked widespread concern and may trigger body dysmorphic disorder (12). The “Snapchat dysmorphia” could be the SNS version of the body dysmorphic disorder diagnosis found in the DSM-5 (13). The “Snapchat dysmorphia” could be applied to one or more perceived defects in physical appearance and be caused by the use of some social medium and some photo retouching software (14). The photo retouching addiction develops gradually. The development of photo retouching addiction essentially is a process characterized by a repeated inability despite significant negative consequences with producing pleasure and escaping from internal discomfort (15). Fu and Luo (16) interviewed some photo retouching addicts and found that photo retouching addicts experienced significant changes in cognitive, behavioral and psychological domains before and after photo retouching behaviors.

At present, there are few scales related to photo retouching addiction for social internet users. According to systematic review of photo retouching behaviors (17), two related photo retouching scales can be seen as follows: (1) The photo modify scale (PMS) by McLean et al. (18) consisted of 10 items used to measure the extent to which social internet users retouched photos before uploading them to social media. Examples of items included issues related to using filters, skin optimization, weight loss, etc. to make a person’s appearance look better. The answer was recorded using a 5-point Likert scale, with a total score ranging from 10 to 50. A high rating indicated a high level of retouching skills for photos. The PMS had a high reliability and a satisfactory validity in the population. (2) The selective self-presentation scale (SSS) by Fox and Vendemia (1) included three items related to photo retouching behaviors: cropping photos, adding filters to photos, and using some retouching software to retouch photos and then uploading them to social media. Social internet users used a 5-point Likert scale to indicate the frequency of using each retouching technique, with higher scores indicating more frequent photo retouching behaviors. The SSS had a high reliability and a satisfactory validity for social internet users.

In the past 5 years, research on photo retouching addiction has included both experimental and cross-sectional methods (17). The experimental method mainly explored the relationship between photo retouching behaviors and body concerns. The experimental method included two different behavioral designs. The first behavioral design measured changes in individual emotions, satisfaction, etc. of social internet users before and after engaging in photo retouching behaviors (19–23). The second behavioral design was to measure the individual emotional and satisfactory changes of social internet users before and after viewing the retouched photos (24). The cross-sectional method mainly focused on the relationship between photo retouching behaviors and body image, body dissatisfaction, and body modification, etc.

However, there are still three shortcomings in previous research as follows: (1) The existing addiction assessment scales for photo retouching behaviors are all quantitative studies on the process of photo retouching behaviors, and have not been extended to study the accompanying emotional disorders or uncontrolled behaviors. (2) As for the research on photo retouching addiction in the past 5 years, more studies focus on the relationship between photo retouching behaviors and body concerns and few studies have connected photo retouching addiction or photo retouching behaviors with social appearance anxiety. (3) There are few studies on photo retouching behaviors that have analyzed the differences of demographic variables such as sex, age, marital status, etc. between photo retouching addiction and social appearance anxiety.

Based on the shortcomings of previous research in three aspects, this study has three objectives as follows: Firstly, the photo retouching addiction scale (PRAS) is reconstructed and developed by self-made method. The developed PRAS includes emotional disorders or uncontrolled behaviors associated with photo retouching behaviors, and are tested for its reliability and validity. The PRAS can be used as a psychological measurement scale to measure photo retouching addiction, providing a measurement basis for photo retouching behaviors of social internet users. Secondly, based on the social appearance anxiety scale (SAAS) (25), a cross-sectional study is used to explore whether there is a correlation between photo retouching addiction and social appearance anxiety. Last but not least, this paper explores whether there are some differences of sex, age, and marital status between photo retouching addiction and social appearance anxiety.

The development of the PRAS is based on three research foundations: (1) Interaction of Person-Affect-Cognition-Execution (I-PACE) mode is used to construct the theoretical foundation of development of the PRAS for social internet users. According to I-PACE mode, it can be observed that photo retouching addicts exhibit significant cognitive, behavioral, and psychological changes before and after their photo retouching behaviors (16). (2) The Selfie Addiction Scale (SAS) (26) is used as a reference for PRAS about body perception, withdrawal tolerance, narcissistic behavior, compulsive behavior, positive expectations, a total of five dimensions. (3) The corresponding interview content of 30 photo retouching addicts about their investing in photographs by technology life history research in media society is used as a reference. Otherwise, the reference to the definition and implications about addiction (15) is used to study photo retouching behaviors.

In summary, the research hypothesis is as follows:

*H1*: The developed PRAS has good reliability and validity for social internet users, which can meet the requirements of measurement.

*H2*: The photo retouching addiction is significantly positively correlated with social appearance anxiety. The total score of PRAS is significantly correlated with the total score of SAAS, and the score of each subscale of PRAS is also significantly correlated with the total score of SAAS.

*H3*: The demographic variables such as sex, age and marital status have a moderating effect in the relationship between photo retouching addiction and social appearance anxiety.

## 2 Methods

### 2.1 Participants

Using a cross-sectional design, 311 participants were randomly selected from social media (WeChat, WeiBo, and Xiaohongshu etc.). These participants were surveyed about PRAS and SAAS in the form of online questionnaire. Before the formal investigation, anonymity was also processed with the consent of the participants, which meets ethical requirements. Informed consent from all participants was obtained. All participants were informed that there were no obstacles to their participation, and that the participants were willing to participate. This study was approved by the South China Normal University (SCNU) research ethics board (Institutional Review Board) who approved the experiments including any relevant details and confirmed that all experiments were performed in accordance with relevant guidelines and regulations.

The participants could not skip any survey items and all answers must be given. The initial participants were 360 people. If the data of participants’ questionnaire responses could not meet the requirements such as explicitly regular answers, careless answers, obvious logical errors in answers, etc., it would be removed, resulting in the retention of 311 participants. The results of the descriptive analysis of the sample data for social internet users were shown in Table 1.

**Table 1:** Descriptive analysis of the sample data for social internet users.

Demographic characteristics	Group	Number of cases	Percentage
	Total	311	100%
Sex	Female	220	29.3%
	Male	91	70.7%
Age	Under 18 years old	33	10.6%
	18–25	90	28.9%
	26–32	127	40.8%
	Over 32 years old	61	19.6%
Marital status	Unmarried	187	60.1%
	Married	124	39.9%

According to Table 1, the total sample of social internet users was 311, of which female 70.7% and male 29.3%; aged under 18 years old (10.6%), aged 18–25 (28.9%), aged 26–32 (40.8%), aged over 32 years old (19.6%); unmarried 60.1% and married 39.9%.

### 2.2 Tools

#### 2.2.1 Photo retouching addiction scale (PRAS)

The formal developed PRAS for social internet users consists of 16 items in five dimensions named as emotional need, withdrawal tolerance, narcissistic behavior, compulsive behavior, and environmental promotion. There are five degrees in the Likert type scale (1 = strongly disagree, 5 = strongly agree), with a total score of 16–80. The development process of the PRAS is as follows:

Firstly, determine the dimensions. The I-PACE model (27), the SAS (26), and the interview data of 30 participants were refereed to determine the dimensions of PRAS. The “emotional need, withdrawal tolerance, narcissistic behavior, compulsive behavior, and environmental promotion” were the final dimension that has been determined.

Secondly, prepare the items. The SAS can be used as the basis for the development of PRAS. The initial 30 items about photo retouching addiction were compiled, and three graduate students were invited to conduct the content validity analysis of a total of 30 items in five dimensions. After the content validity analysis, only 24 items were obtained.

Thirdly, examine the samples. Sample data were obtained by testing 311 participants through internet media. For the data of 311 participants, the almost half was for exploratory factor analysis (EFA) and the other almost half was for confirmatory factors analysis (CFA) to verify its validity. Simultaneously, the aggregation validity and distinguish validity were also analyzed. Otherwise, the overall scale reliability and the reliability of each subscale were also analyzed.

Finally, form formal scale. After performing the above steps, the 16 formal items were finally determined to be retained and were divided into five dimensions. There were 19 items in the PRAS, among which the first three items were demographic information including sex, age, and marital status and the last 16 items were 16 questions about PRAS.

#### 2.2.2 Social appearance anxiety scale (SAAS)

The SAAS developed by Hart et al. (25) measured the level of anxiety negatively rated by others for a person’s overall appearance (including body shape). The scale consisted of 16 items (eg, “when I talk to others, I feel nervous because of my appearance” “I feel uncomfortable when others notice my flaws on the outside”), and participants indicated individual behavioral characteristics on the Likert scale from 1 (strongly disagree) to 5 (strongly agree). In this study, the SAAS fit the sample data well, and the fitting indicators of confirmatory factor analysis were as follows: χ^2^(301) = 311.89; χ^2^/*df* = 2.99; CFI = 0.99; TLI = 0.99; RMSEA = 0.061; SRMR = 0.051. Each item of SAAS also showed high factor loading. For the Cronbach’s Alpha result, the reliability of SAAS was 0.94. These results indicated that the SAAS had good reliability and validity for social internet users (28, 29).

### 2.3 Data processing

The questionnaire consists of three parts: The first part is social demographic characteristics (sex, age, marital status), with 3 items (from item 1 to item 3) as follows: (1) item 1: sex (male; female); (2) item 2: age (under 18 years old; 18–25; 26–32; over 32 years old); (3) item 3: marital status (unmarried; married). The second part is the PRAS, which has 16 items (from item 4 to item 19); and the developed PRAS from item 1 to item 19 can be found in the Appendix of this article. The third part is the SAAS, which has 16 items (from item 20 to item 35). The total number of items is 35. The EFA was used Spss28 and the CFA was used Amos28 to obtain the structure validity and aggregation validity of PRAS for social internet users. To test the overall reliability and dimensional reliability of the scale of PRAS and SAAS for social internet users, Spss28 was used.

## 3 Results

### 3.1 The performance of developed PRAS

To test the validity of PRAS, the factor analysis was conducted to determine its validity and factor composition, so as to test the reliability later. Almost half of the samples were randomly selected with Spss28 for EFA and the other almost half for CFA with Amos28. The 311 participants were randomly divided into two halves (approximate), half was 149 persons as sample 1 for EFA, and the other half was 162 participants as sample 2 for CFA.

Using KMO and Bartlett’s test of sphericity for sample 1 with Spss28 software, KMO value was 0.804 and *p*-value of sphere test was *p* < 0.001, which indicated that sample 1 was suitable for factor analysis. In this study, the principal component oblique rotation was used for factor extraction on sample 1, five factors (characteristic values greater than 1), and the percentage of variance after rotation was 17.728, 14.153, 14.029, 13.497, 12.861%, and the cumulative sum of squares was 72.268%. According to the correlation of the factors and each item, the five factors were named as emotional need, withdrawal tolerance, narcissistic behavior, compulsive behavior, and environmental promotion. The dimensions and factor loading of PRAS were shown in Table 2.

**Table 2:** The dimension and factor loading of PRAS.

Item	Emotional need	Withdrawal tolerance	Narcissistic behavior	Compulsive behavior	Environmental promotion
Item 4	**0.873**	0.005	0.073	−0.108	0.047
Item 5	**0.780**	0.079	0.035	0.177	0.193
Item 6	**0.801**	0.117	0.149	0.039	0.064
Item 7	**0.810**	0.078	0.145	0.067	0.116
Item 8	0.064	**0.862**	0.065	0.071	0.196
Item 9	0.148	**0.757**	0.087	0.147	0.164
Item 10	0.041	**0.663**	0.260	0.262	0.245
Item 11	0.068	0.036	**0.894**	0.064	0.082
Item 12	0.126	0.213	**0.812**	0.088	0.062
Item 13	0.213	0.101	**0.737**	0.249	0.163
Item 14	0.011	−0.022	0.104	**0.851**	0.212
Item 15	−0.026	0.275	0.138	**0.796**	0.051
Item 16	0.152	0.189	0.122	**0.803**	−0.011
Item 17	0.126	0.196	0.043	−0.026	**0.876**
Item 18	0.161	0.211	0.031	0.135	**0.759**
Item 19	0.099	0.153	0.258	0.155	**0.742**

Bold values indicate that the items belong to the corresponding dimension.

As could be seen from Table 2, the first factor “emotional need” included four items, from item 4 to item 7, whose factor loadings ranged from 0.780 to 0.873. The second factor “withdrawal tolerance” included three items, from item 8 to item 10, whose factor loadings ranged from 0.663 to 0.862. The third factor “narcissistic behavior” included three items, from item 11 to item 13, whose factor loadings ranged from 0.737 to 0.894. The fourth factor “compulsive behavior” included three items, from item 14 to item 16, whose factor loadings ranged from 0.796 to 0.851. The fifth factor “environmental promotion” included three items, from item 17 to item 19, whose factor loadings ranged from 0.742 to 0.876.

The sample 2 was used for CFA by Amos28 software, and the overall fit indicators were shown in Table 3.

**Table 3:** Overall fit coefficient for the confirmatory factor analysis.

χ^2^/*df*	CFI	TLI	RMSEA	SRMR
1. 511	0.975	0.968	0.041	0.031

According to Table 3 (χ^2^/*df* = 1.511 < 3, CFI = 0.975 > 0.90, TLI = 0.968 > 0.90, RMSEA = 0.041 < 0.08, SRMR = 0.031 < 0.08), these fitting indicators indicated good fit of the scale and good structural validity (28, 29).

The sample 2 was used for CFA, and the factor loading was shown in Table 4.

**Table 4:** Factor loading.

Item	Way	Factor	Estimate	AVE	CR
XTQ4	<−	Emotional need	0.864	0.628	0.871
XTQ5	<−	Emotional need	0.789		
XTQ6	<−	Emotional need	0.747		
XTQ7	<−	Emotional need	0.765		
XTQ8	<−	Withdrawal tolerance	0.809	0.573	0.800
XTQ9	<−	Withdrawal tolerance	0.694		
XTQ10	<−	Withdrawal tolerance	0.763		
XTQ11	<−	Narcissistic behavior	0.788	0.532	0.773
XTQ12	<−	Narcissistic behavior	0.705		
XTQ13	<−	Narcissistic behavior	0.691		
XTQ14	<−	Compulsive behavior	0.778	0.518	0.762
XTQ15	<−	Compulsive behavior	0.656		
XTQ16	<−	Compulsive behavior	0.720		
XTHQ17	<−	Environmental promotion	0.871	0.583	0.806
XTHQ18	<−	Environmental promotion	0.726		
XTHQ19	<−	Environmental promotion	0.680		

From Table 4, the loading of each factor was greater than 0.60 and was qualified. The combined reliability CR was greater than 0.70 and the mean variance extraction amount AVE was greater than 0.50, which indicated that there was good aggregate validity for PRAS.

The Cronbach’s Alpha of the overall scale was 0.862, and the Cronbach’s Alpha of each dimension was 0.862 (emotional need), 0.784 (withdrawal tolerance), 0.789 (narcissistic behavior), 0.774 (compulsive behavior), and 0.836 (environmental promotion). The above Cronbach’s Alpha values were greater than 0.770, indicating the internal consistency of all dimensions of the scale was good, so the reliability of the results of this survey was good.

### 3.2 The statistical results of photo retouching addiction and social appearance anxiety

The descriptive statistics of the sample data were shown in Table 5.

**Table 5:** The descriptive analysis of the sample data.

Demographic characteristics	Group	Mean score on the PRAS (standard deviation)	Mean score on the SAAS (standard deviation)
	Total	53.39 (10.63)	46.70 (12.61)
Sex	Female	51.83 (9.72)	46.98 (12.20)
	Male	57.14 (11.77)	46.03 (13.61)
Age	Under 18 years old	48.82 (6.19)	36.52 (10.84)
	18–25	58.21 (12.22)	49.70 (11.61)
	26–32	54.53 (10.20)	50.61 (11.02)
	Over 32 years old	46.36 (4.84)	39.64 (12.28)
Marital status	Unmarried	54.55 (11.97)	50.05 (11.42)
	Married	51.63 (7.92)	42.65 (12.69)

For the 311 participants in Table 5, the range of mean score on the PRAS was 46.36–58.21 and the standard deviation was 4.84–12.22. The range of mean score on the PRAS was broad, but no extreme data appeared. Similarly, the 311participants of Table 5 had the range of 36.52–50.61 for mean score on the SAAS and the standard deviation value range of 10.84–13.61. The range of mean score on the SAAS had a certain breadth, but no extreme data appeared.

**Figure 1:**
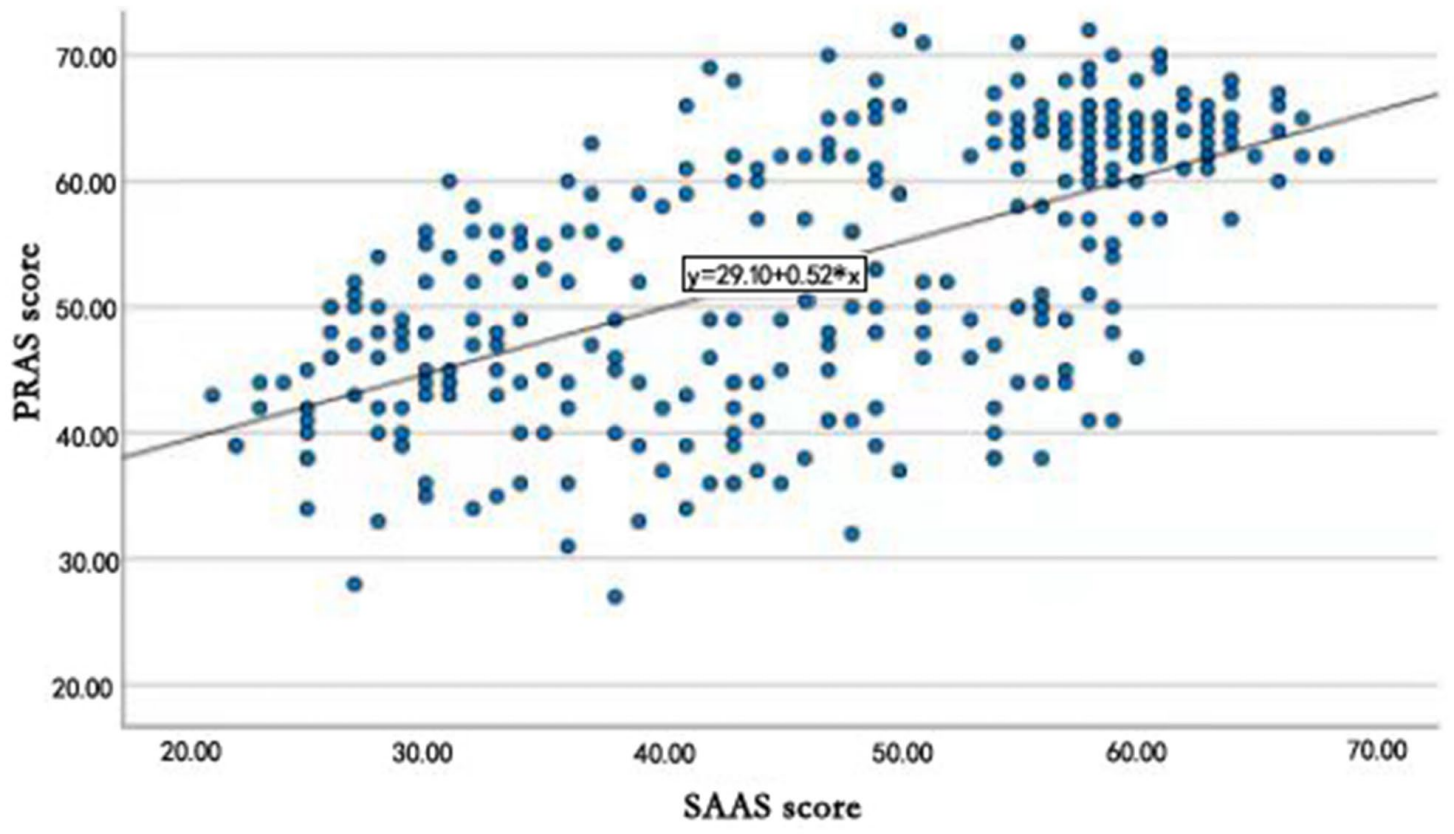
Linear regression analysis.

Social appearance anxiety and photo retouching addiction were significant by Pearson correlation analysis (*p* < 0.001). Social appearance anxiety and the five dimensions of photo retouching addiction scale (emotional need, withdrawal tolerance, narcissistic behavior, compulsive behavior, and environmental promotion) were also significant (*ps* < 0. 001), with the correlation coefficient of 0.717, 0.672, 0.678, 0.618, 0.705. The results of the linear regression analysis were shown in Figure 1.

From Figure 1, linear regression analysis was used to study the relationship between social appearance anxiety (X, SAAS score, independent variable) and photo retouching addiction (Y, PRAS score, dependent variable). As for Figure 1, a straight line according to the distribution of scatter plots could be seen, which indicated that there were good fitting results and the relationship between social appearance anxiety and photo retouching addiction was linear. Significant correlation was shown (*p* < 0.001), with constant of 29.10 and coefficient of 0.52, resulting in the regression equation Y = 29.10 + 0.52X. The *R*^2^ was 0.38.

### 3.3 The effect of demographic variables between photo retouching addiction and social appearance anxiety

The total score of PRAS ranged from 27 to 72, with a mean score of 53.39 and a standard deviation of 10.63. The sample skewness was −0.21 and kurtosis was −1.12, which was consistent the normal distribution. After independent sample t-test, the total score of men was significantly higher than total score of women (*t* = 3.801, *p* = 0.008, *d* = 0.85) and the total score of unmarred population was significantly greater than the total score of married population (*t* = 2.591, *p* = 0.009, *d* = 0.68). By one-way analysis of variance and *post hoc* comparison, the main effect of age was significant (*F* = 21.004, *p* = 0.004, *η*^2^ = 0.15). The total score of participants aged 18–25 years old was significantly higher than those total score of other three age groups, the total score of participants aged 26–32 years old was significantly higher than those total scores of participants aged under 18 years old and those over 32 years old, but there was no significant difference between those total scores of participants aged under 18 years old and over 32 years old.

The total sample of this survey was 311 participants, and the total score of SAAS ranged from 21 to 68, with an average score of 46.70 and standard deviation of 12.61; the skewness was −0.27 and the kurtosis was −1.22, which met the normal distribution. After independent sample t-test, the total score of men was not significantly different from the total score of women (*t* = 0.600, *p* = 0.549, *d* = 0.01), and the total score of unmarried people was significantly higher than the total score of married people (*t* = 6.070, *p* < 0.001, *d* = 1.01). By one-way analysis of variance and *post hoc* test, the main effect of age was significant (*F* = 23.526, *p* = 0.002, *η*^2^ = 0.16). There was no difference in the total score between participants aged 18–25 years old and participants aged 26–32 years old, but both were significantly higher than under 18 years old and over 32 years old (*p* < 0.001).

To explore whether demographic characteristics played a moderating role between photo retouching addiction and social appearance anxiety, the sex, age and marital status were stratified to analyze. Especially, taking a stratified regression of age as a moderating variable, the age was converted into a dummy variable with values 0 and 1, ended with a total of three age dummy variables, and then shaped two models with this. In model 1, the age dummy variables and standardized social appearance anxiety scores were included as independent variables and standardized photo retouching addiction scores were included as dependent variables. On the basis of model 1, the interaction term of the age (dummy variable) and the standardized social anxiety score were added as the independent variable to shape model 2. Linear regression was performed with Spss28 to obtain the determination coefficient *R*^2^_1_, *R*^2^_2_ and Δ*R*^2^. The ΔR^2^ was used to determine whether age had a significant moderating effect. The stratified regression for the moderating effects of sex and marital status was similar to the above age. All the results of sex, age and marital status were shown in Table 6.

**Table 6:** Analysis of the moderating effects.

Variables	Model	*R*	*R* ^2^	Δ*R*^2^	Δ*F*	*p*
Sex	1	0.666	0.443	0.443	122.522	<0.001
	2	0.685	0.470	0.027	15.534	<0.001
Age	1	0.658	0.433	0.433	58.521	<0.001
	2	0.717	0.514	0.081	16.856	<0.001
Marital status	1	0.621	0.386	0.386	96.839	<0.001
	2	0.636	0.405	0.019	9.703	0.002

From Table 6, the moderating effects of sex, age, and marital status were all significant. Age had the largest effect (8.1%), followed by sex (2.7%) and the minimal by marital status (1.9%).

## 4 Discussions

### 4.1 The relationship between selfie addiction and photo retouching addiction

There is a certain correlation between selfie addiction and photo retouching addiction for the following reasons: (1) the photo retouching addiction cannot be separated from selfie behavior. Selfie addiction is a prerequisite behavior for photo retouching addiction, and photo retouching behaviors need to rely on selfie behaviors to achieve it. (2) The selfie addiction and photo retouching addiction demonstrate a dependence on social media for social internet users.

However, there is a relatively independence between selfie addiction and photo retouching addiction on social media for social internet users as follows: on the one hand, selfie addiction patients and photo retouching addiction patients do not completely overlap; on the other hand, selfie addicts may not be photo retouching addicts, and photo retouching addicts may not be selfie addicts either. Selfie addiction and photo retouching addiction may also present different directions in terms of harm. Selfie addiction may lead to accidental injuries and deaths due to dangerous shooting behavior, while photo retouching addiction may extend outward to appearance “optimization” (plastic surgery) and body phobia disorder due to an individual’s excessive focus on appearance defects. Dokur et al. (30) conducted a study on selfie related injuries and deaths and found that selfie related injuries and deaths had been increasing in recent years, especially among adolescents and young adults who were more susceptible to selfie related injuries and deaths.

From the perspective of “dependence” about the relationship between selfie addiction and photo retouching addiction, the PRAS could largely draw on some dimensions and items of the SAS. This study drew inspiration from the nine dimensions of the SAS. Based on their relationship between PRAS and SAS, the PRAS drew on the five dimensions of the SAS (body awareness, withdrawal tolerance, narcissistic behavior, compulsive behavior, positive expectations). Correspondingly, it gradually formed the five dimensions of the PRAS for social internet users, namely emotional need, withdrawal tolerance, narcissistic behavior, compulsive behavior, and environmental promotion.

From the perspective of “independence” about the relationship between selfie addiction and photo retouching addiction, dimensions and questions suitable for photo retouching addiction had also been developed, which was different from selfie addiction. Based on the “independence” and “dependence” between selfie addiction and photo retouching addiction, about half of the items of PRAS were referred to the SAS, but the other half of the items of PRAS were not related to SAS and were self-developed in this paper.

### 4.2 The relationship between I-PACE model and photo retouching addiction

The I-PACE model is proposed based on the topic of internet addiction, which is a theoretical framework. It believes that specific internet use disorders are the result of the interaction of inducing factors (such as neurobiological and psychological composition), regulating factors (such as coping styles and internet related cognitive bias), and mediating factors (such as emotional and cognitive responses to situational triggers). Accompanied by reduced executive function, the process of conditioned reflex may strengthen these associations in the process of addiction (27). The I-PACE model integrates the findings and theoretical assumptions of other research fields such as self-objectification theory (31) and social comparison theory (32). The I-PACE model refers to the known concepts in the study of substance dependence (e.g., DSM-5 criteria or behavioral addiction models), which is consistent with the idea of classifying internet use disorder and other behavioral addiction together with substance use disorder as addictive behavior (33).

The I-PACE model can effectively explain the various components of photo retouching addiction in this study. The I-PACE model includes cognitive bias, behavioral response, psychological composition, etc., while the photo retouching addiction is highly consistent with the I-PACE model based on internet addiction. It can be seen from this that the photo retouching addiction belongs to internet addiction and is a kind of internet addiction. The I-PACE model can effectively explain the vast majority of the main components of photo retouching addiction. According to the I-PACE model, the photo retouching behaviors for photo retouching addicts are mainly based on cognitive needs, behavioral needs, and psychological needs, which is also supported by interview data from 30 photo retouching addicts.

The use of the I-PACE model in this study is reasonable. Based on the content of this study, it can be found that the I-PACE model effectively interprets the various dimensions of the PRAS, namely emotional need, withdrawal tolerance, narcissistic behavior, compulsive behavior, and environmental promotion. Among them, withdrawal tolerance is a triggering factor (such as neurophysiology and psychological composition), environmental promotion is a regulating factor (such as coping style and internet related cognitive bias), emotional need is a mediating factor (such as the result of the interaction of emotion and cognitive response to situational triggers), narcissistic behaviors and compulsive behaviors are executive consequences (if emotion, environment and emotion are well handled, executive function will be improved). In summary, the I-PACE model can effectively explain the dynamic process of photo retouching addiction and provide an excellent theoretical foundation for the development of the PRAS.

### 4.3 The quality analysis of PRAS

Some literature suggested that selfie addiction and photo retouching addiction had a certain correlation (19, 34–36), but there were also considerable differences (20, 21, 37, 38). Therefore, there is independent research significance for photo retouching addiction (9, 11, 17). The PRAS in this study can to a large extent draw lessons from some of the dimensions and items of SAS. In this paper, about half of the items of PRAS are related to SAS.

Five dimensions of PRAS “emotional need, withdrawal tolerance, narcissistic behavior, compulsive behavior, and environmental promotion” are corresponding to five dimensions of SAS “body perception, withdrawal tolerance, narcissistic behavior, compulsive behavior, positive expectations” respectively (26). The first dimension “emotional need” indicates that social internet users retouch photos from emotional needs and they may experience emotions such as anxiety or depression if they do not retouch their photos. The second dimension “withdrawal tolerance” indicates that social internet users may experience withdrawal symptoms due to their tolerance for photo retouching behaviors. The third dimension “narcissistic behavior” indicates that social internet users have developed narcissism due to feeling good about themselves and leading to photo retouching behaviors. The fourth dimension “compulsive behavior” indicates that social internet users have compulsive behaviors due to self-loss of control which is a symptom of addiction. The fifth dimension “environmental promotion” indicates that social internet users have continuous photo retouching behaviors due to environmental factors with “follower effect” and “conformity effect.”

The factor loadings of all 16 items are higher than 0.663, which indicates a strong correlation between the item and the factor. According to the PRAS, its total score was 16–80 points. In terms of the quality of the items, the total score range of 311 participants was 46.36–58.21 and the standard deviation was 4.84–12.22. The range of the total score had a certain breadth, but there was no extreme data, indicating that the sample was highly representative. The items’ loading of all the factors was higher than 0.663, indicating that the items were highly related to the factors, and the common degree value of each factor was higher than 0.5, meaning that there was a strong correlation between the items and the factors. The items in the factors did not appear in other factors with high correlation, which indicated that the items in the factors were obviously discriminated and supported a good foundation for the higher structural validity. It also showed that the factor loading of all the items was good. Therefore, all these items of developed PRAS were good.

In terms of scale validity, the indicators of the confirmatory factor analysis (χ^2^/*df* < 5, CFI > 0.90, TLI > 0.90, RMSEA < 0.08, SRMR < 0.08) showed good structural validity, indicating that the empirical data well fit the five dimensional structures of the exploratory factor analysis (28, 29). The correlation was significant among the factors, and the correlation coefficient was also lower than the square root of AVE, and there was no high correlation between the factors, indicating that the discrimination validity of the whole scale is good. Therefore, it could be seen that the model had good structural validity, aggregate validity and discriminatory validity. Therefore, this indicated that the developed PRAS had good validity.

In terms of scale reliability, the Cronbach’s Alpha of the developed PRAS was 0.862, with dimensions of 0.862 (emotional need), 0.784 (withdrawal tolerance), 0.789 (narcissistic behavior), 0.774 (compulsive behavior), and 0.836 (environmental promotion), indicating good overall and dimensional reliability. In summary, from the perspective of item quality, reliability and validity, the developed PRAS had a reasonable structure with good reliability and validity, and sufficient theoretical basis for reference.

### 4.4 The relationship between photo retouching addiction and social appearance anxiety

Vandenbosch et al., (39) found that taking and editing (but not posting) selfies resulted in negative effects on body image. In this study, social appearance anxiety and photo retouching addiction were significantly associated after Pearson correlation analysis. Besides, social appearance anxiety and the five dimensions of the PRAS (emotional need, withdrawal tolerance, narcissistic behavior, compulsive behavior, and environmental promotion) were all significantly associated, indicating that the photo retouching addiction was significantly positively associated with social appearance anxiety. Further, linear regression analysis showed that social appearance anxiety (independent variable) and photo retouching addiction (dependent variable) could fit a straight line according to the scatter plot distribution, which indicated this trend that the higher the social appearance anxiety was, the higher the photo retouching addiction was, and vice versa. Frequent exposure to appearance ideal social media is associated with body dissatisfaction. Commercial and peer social media literacy would protect against the negative impact of exposure to social media appearance ideal images on young adults’ body image (40).

In this paper, the *R*^2^ was 0.38, which indicated social appearance anxiety was a very important factor of photo retouching addiction. There are two main reasons for this: On the one hand, because of self-image management that gives people more love for beauty, people are more concerned about the impression of their own appearance on others in current society. As a result, more and more people concern about the presentation of beauty on the internet. On the other hand, there may be a mentality of comparison, which means that people like to compare themselves. If others make themselves more beautiful, people would also make themselves more beautiful, and some people even believe in this.

From some literatures (7, 20, 21, 38, 41, 42), it can be concluded that social appearance anxiety and photo retouching addiction may have a significant connection. Chang et al. (41) found an indirect relationship between photo retouching addiction and body self-esteem, mediated by physical comparison of peers. Teran et al. (42) found an association between retting and appearance evaluation in American adolescent women, both significantly intermediate in self-objectification. Sun (38) found that the degree of photo retouching addiction was positively correlated with facial dissatisfaction in young adult women in China. However, the correlation between the self-materialization was only significant in the test of structural equation model. Parsa et al. (20) found that photo retouching addiction could increase this dissatisfaction and affect the subjects’ intention of plastic surgery from the perspective of experimental research.

### 4.5 The roles of demographic variables between photo retouching addiction and social appearance anxiety

From the mean score on the PRAS, the total score ranged from 16 to 80 points, and the median was 48, except for over 32 years old (46.36), all were higher than 48 points, indicating that most of the subjects had a certain degree of photo retouching addiction. Similarly, from the mean score on the SAAS, the total score ranged from 16 to 80, and the regular model was 41, higher than 41 except for under 18 years old and over 32 years old (39.64), indicating that most of the subjects had a certain degree of social appearance anxiety.

These scores of PRAS fit to a normal distribution, indicating that independent sample t-test or ANOVA can be used. Men have significantly more addiction scores than women, indicating that men are more inclined to acquire photo retouching addiction, which is different from ordinary people who understand that women love dressing. One possible reason is that men on the internet are no less “retouching” or “disguise” than women. In women only, commercial-social media literacy moderated the negative effect of exposure, independent of internalization or body comparison (40). The intensity of social media use may influence body image outcomes in young adult women (43). In terms of age, the addiction scores were significantly higher in participants aged 18–25 than in the other three age groups, indicating that this age group is the main population and needs particular attention. The scores of the unmarried are significantly higher than those who are married, indicating that unmarried people often have to do photo retouching because they may attract the attention of the opposite sex as soon as possible.

These scores of SAAS fit to a normal distribution, indicating that an independent sample t-test or ANOVA can be performed. The scores of SAAS for men are not significantly different from women, but the scores of SAAS for unmarried people are significantly greater than those of married people. In terms of age, participants aged 18–32 are more likely to lead to social appearance anxiety, which are significantly higher than those of participants under 18 years old and over 32 years old, indicating that this group needs special attention. As adolescents’ negative body image perception and social media addiction increased, social media consciousness about appearance increased (44).

The purpose of stratified regression of sex, age and marital status can be to intuitively isolate the effects of these demographic variables. From the results, the moderating effects of sex, age and marital status are all significant. The results of the moderating effect analysis further verified the results of the independent sample t-test and ANOVA, made the correspondence test well, and explained the effect size generated by sex, age and marital status. From the results, the age effect was largest at 8.1% (0.081), followed by sex at 2.7% (0.027), and the minimal was marital status at 1.9% (0.019).

### 4.6 Limitations

Firstly, the samples can be further expanded in the future research and the sample collection cannot be restricted from online collection. The research cannot be only limited to self-reported questionnaire survey, and it’s also possible to carry out by experimental method. Secondly, this is a cross-sectional study; whether these conclusions can be applied to longitudinal studies is not clear. Therefore, future studies may be longitudinal in nature to strictly confirm the causal relationships among these variables. Thirdly, this study is limited to demographic variables such as sex, age, and marital status, and other demographic variables can also be explored, such as educational background, occupation, frequency of social media usage, social support, and geographical environment etc. Last but not least, the differences in sociocultural background are the possible limitations for the generalizability of the findings. There is no discussion of how cultural context may influence the prevalence or expression of photo retouching behaviors.

## 5 Conclusion

1. The developed photo retouching addiction scale (PRAS) includes five dimensions named as emotional need, withdrawal tolerance, narcissistic behavior, compulsive behavior, and environmental promotion and has good reliability and validity (Cronbach’s Alpha = 0.862, χ^2^/*df* = 1.511, CFI = 0.975, TLI = 0.968, RMSEA = 0.041, SRMR = 0.031), which can be recommended to use in practice.
2. The photo retouching addiction is significantly and positively correlated with social appearance anxiety. Social appearance anxiety has a significant impact on photo retouching addiction, which indicates this trend that the higher the social appearance anxiety is, the higher the photo retouching addiction is.
3. Sex, age and marital status have a significant impact in the relationship between photo retouching addiction and social appearance anxiety. Age has the largest effect (8.1%), followed by sex (2.7%) and the minimal by marital status (1.9%). The attention should be paid to these demographic variables between photo retouching addiction and social appearance anxiety.

## Data Availability

The datasets presented in this study can be found in online repositories. The names of the repository/repositories and accession number(s) can be found in the article/Supplementary material.

## Appendix

### Photo Retouching Addiction Scale (PRAS)

Dear friend, there are some questions related to photo retouching addiction. Please answer them according to your actual situation. For the demographic variables in item 1 to item 3, please answer according to the actual situation in ☐. For item 4 to item 19, please answer according to your actual level, which is divided into five levels, with 1 indicating strongly disagree; 2 indicating disagree; 3 indicating basic agree; 4 indicating agree; 5 indicating strongly agree. Please answer one of 1-5 in ☐.

**Table:**

1. Sex: Male ☐, Female ☐.	
2. Age: Under 18 years old ☐, 18–25 years old ☐, 18–25 years old ☐, Over 32 years old ☐.	
3. Marital status: Unmarried ☐, Married ☐.	
4. I cannot wait to retouch photos and can hardly wait for a moment.	☐
5. In the face of the photos, I am unable to restrain the impulse to retouch photos.	☐
6. After retouching and posting photos on social media, it makes me feel comfortable.	☐
7. I am very focused and immersed when retouching photos, otherwise it will make me anxious.	☐
8. I can tolerate longer than expected retouching time for better results.	☐
9. To achieve perfection, I sent out retouched photos several times and then revised them back.	☐
10. If the photos don’t turn out well, I will retouch them and can tolerate this behavior.	☐
11. For the sake of my beautiful image, I am very focused and immersed when retouching.	☐
12. I feel good about myself and it is necessary to retouch photos in the social circle.	☐
13. If I don't retouch photos in the social circle, it may ruin my good daily image.	☐
14. To achieve better results, I will unconsciously retouch photos repeatedly.	☐
15. The act of retouching photos makes me feel emotionally forced and difficult to control.	☐
16. I am willing to keep retouching photos, even if I can't stop.	☐
17. Everyone is retouching photos, and I also have this strong desire.	☐
18. If I don't retouch photos, I will feel like I'm losing out.	☐
19. I think that people with better looking photos have more social advantages in the social circle.	☐
